# Use of convalescent plasma therapy in hospitalised adult patients with non-critical COVID-19: a focus on the elderly from Hungary

**DOI:** 10.1007/s11357-022-00683-4

**Published:** 2022-11-11

**Authors:** Noemi Kiss-Dala, Balint Gergely Szabo, Botond Lakatos, Marienn Reti, Janos Szlavik, Istvan Valyi-Nagy

**Affiliations:** 1 School of PhD Studies, Semmelweis University, H-1085 Ulloi Ut 26, Budapest, Hungary; 2 South Pest Central Hospital, National Institute of Haematology and Infectious Diseases, Szent Laszlo Campus, H-1097 Albert Florian Ut 5-7., Budapest, Hungary

**Keywords:** SARS-CoV-2, COVID-19, Coronavirus disease, Convalescent plasma therapy, Elderly

## Abstract

Convalescent plasma therapy might be a feasible option for treatment of novel infections. During the early phases of the severe acute respiratory syndrome coronavirus-2 (SARS-CoV-2) pandemic, several promising results were published with convalescent plasma therapy, followed by more disappointing findings of randomised controlled trials. In our single-centre, open-label, prospective, cohort study, we assessed the findings of 180 patients treated with convalescent plasma during the first four waves of the pandemic in Hungary. The primary outcome was all-cause mortality; secondary outcomes were clinical improvement and need for intensive care unit admission by day 28. Subgroup analysis comparing elderly and non-elderly (less than 65 years of age) was performed. Twenty (11.4%) patients died by day 28, at significantly higher rates in the elderly subgroup (3 vs. 17, *p* < 0.01). One hundred twenty-eight (72.7%) patients showed clinical improvement, and 15 (8.5%) were transferred to the intensive care unit until day 28. Non-elderly patients showed clinical improvement by day 28 in significantly higher rates (improvement 74 vs. 54, no improvement 15 vs. 11, worsening or death 4 vs. 18 patients, *p* < 0.01). In conclusion, we found similar clinical outcome results as randomised controlled trials, and the impact of risk factors for unfavourable clinical outcomes among patients in the elderly population.

## Introduction

At the beginning of 2020, a new pandemic started across the globe, caused by the severe acute respiratory syndrome coronavirus-2 (SARS-CoV-2), affecting millions of people and causing deaths in immense numbers worldwide. Since then, health care systems have experienced a serious pressure, facing the novel respiratory illness called coronavirus disease 2019 (COVID-19). Naturally, the pandemic encouraged the scientific society to search for effective treatment strategies as soon as possible.

SARS-CoV-2 is a positive-sense, single-stranded RNA virus belonging to the family *Coronaviridae*. Most COVID-19 cases are asymptomatic or have a mild respiratory disease course, but, in some patients, it manifests as severe pneumonia, even requiring intensive care unit admission and mechanical ventilation, or leading to death [1]. In the early phase of an epidemic caused by a novel infectious agent, passive immunotherapy can be a feasible treatment option. Passive immunotherapy with convalescent plasma dates back to the late nineteenth century, when it was first used as an effective therapy for diphtheria. Since then, it has been applied in several disease outbreaks, such as the 1918 Spanish flu pandemic, the global spread of 2005 H5N1 avian influenza [2, 3], the 2009 H1N1 influenza pandemic [4], the 2003 SARS [5, 6] and 2013 Middle East respiratory syndrome coronavirus (MERS) epidemics [7], and the 2014 West African Ebola outbreaks [8, 9], with promising clinical outcomes [10, 11]. Most of these studies concluded that immunotherapy with convalescent plasma may be effective early in the disease course, explained by the early viral phase of the diseases and the delayed immune responses. Besides the passive immunisation mechanism, convalescent plasma therapy may have an immunomodulatory effect as well [12], including direct virus neutralisation, control of an overreacting immune response (i.e. cytokine storm, helper T-lymphocyte functions, complement activation) and immunomodulation of a hypercoagulable state. Lastly, convalescent plasma can be obtained from survivors in a relatively easy manner even in low-income countries, providing the opportunity to treat acutely infected patients with specific antibodies.

At the early phases of the SARS-CoV-2 pandemic, experiences with plasma therapy were only available from previous disease outbreaks. Our aim was to assess the clinical use of convalescent plasma therapy in non-critical hospitalised adult COVID-19 patients, with a specialised focus on the elderly populations at our centre.

## Methods

### Study design and setting

A single-centre, open-label, prospective, observational study was carried out among a cohort of hospitalised adult COVID-19 patients receiving convalescent plasma therapy between 1 April 2020–31 December 2021 at the South Pest Central Hospital, National Institute of Haematology and Infectious Diseases (Budapest, Hungary), the national referral centre with more than 150 dedicated beds for COVID-19 patients during the pandemic. All patients gave informed consent for anonymised data collection and processing. The study was in accordance with the Helsinki Declaration and national ethical standards. The study protocol, as part of the CONTRAST (**CO**mparing **N**ovel **TR**eatment Strategies **A**gainst **S**ARS-CoV-**T**wo) clinical trial, has been approved by Institutional Review Board of South Pest Central Hospital, National Institute of Haematology and Infectious Diseases (EB-14/2020) and the Scientific and Research Ethics Committee of the Hungarian National Medical Scientific Council (ETT-TUKEB IV/3937–1/2020/EKU).

### Patient eligibility and participant selection

All symptomatic adult patients with respiratory SARS-CoV-2 PCR positivity hospitalised at our centre during the first four waves of the COVID-19 pandemic in Hungary were eligible for inclusion. Patient enrollment was performed consecutively during daily on-site and real-time visits by attending physicians. All patients receiving COVID-19 convalescent plasma (CCP) therapy for COVID-19 were included in the final cohort; patients receiving other medications for COVID-19 were not excluded. Anonymised data of included patients were collected from electronic medical charts and paper-based documentation into a standardised case report form.

### Definitions

Mild COVID-19 at baseline was defined as categories 1 to 3 on the World Health Organisation (WHO) Clinical Progression Scale (CPS), moderate disease as categories 4 and 5, severe disease as category 6 [https://www.who.int/docs/default-source/documents/emergencies/minimalcoreoutcomemeasure.pdf]. Since in the original WHO-CPS description the application of the different oxygen supplements are not detailed, we used the flow rate of 12 l pro minute for the distinction between mask or nasal prongs supplementation in category 5 and non-invasive ventilation or high-flow oxygen administration in category 6, as another study suggested [13].

Risk factors for progression to severe or critical COVID-19 disease were defined as > 60 year of age, resident of a long-term care facility, underlying severe or chronic comorbidities (essential hypertension, obesity, diabetes mellitus, chronic cardiovascular, cerebrovascular, kidney disease, chronic obstructive pulmonary disease, chronic immunosuppression including active haematological and oncological malignancies, congenital immunodeficiencies, asplenia, uncontrolled HIV infection, solid organ or haematopoietic stem-cell transplant recipient, chemotherapy or other immunosuppressant therapy in the previous 6 months, systemic corticosteroid therapy ≥ 20 mg/die prednisolone equivalent dose for ≥ 2 weeks, systemic autoimmune diseases, hepatic cirrhosis and chronic alcohol abuse.

Date of symptom onset was defined as the day of the first recognition of any COVID-19 attributable symptom, reported by the patient or caregiver. When the patient remained asymptomatic or where no relevant data was available, the date of symptom onset was marked as the day of the first positive respiratory SARS-CoV-2 PCR or first day of recognition of any COVID-19 attributable symptom by a health care professional. The day of symptom onset and the day of receiving the first unit of convalescent plasma were considered as day 0 during specified statistical analyses.

### Administration of COVID-19 convalescent plasma and follow-up of COVID-19 patients

Indications for COVID-19 convalescent plasma (CCP) therapy were severe COVID-19 disease showing no clinical improvement to other treatments within 72 h of therapy initiation, and mild to moderate COVID-19 with high risk for progression to severe or critical disease. Contraindications for CCP were documented total serum IgA or haptoglobin deficiency, documented severe allergic reaction related to the use of blood products, development of fluid overload and lack of informed consent. Each patient received at least one unit of ABO and Rh blood group compatible CCP over a period of ≥ 1 h. Patients were monitored closely for adverse events until 12 h after the CCP was administered.

The clinical status of the patients was monitored daily by the attending physicians until hospital discharge, intensive care unit admittance or death. Chest imaging (computer tomography or chest X-ray if computer tomography was not feasible) was performed in 24 h from admission, and repeated every 7 days or when clinical deterioration was observed. Supplemental oxygen was administered via low-flow nasal cannula, high-flow nasal cannula or Venturi facemask. Clinical status and oxygen supplementation rate were evaluated on days 0, 3, 5, 7, 10, 14 and 28 from the initiation of CCP therapy. Data regarding length of hospital stay, length of intensive care unit (ICU) stay, need for ICU admittance and time to ICU admittance were collected until hospital discharge or death. Post-discharge follow-up until a total of 28 days since index hospital admission was done by clinical outpatient visits, telephone calls or the *National eHealth Infrastructure*.

### Study outcomes and statistical analysis

The primary outcome was all-cause in-hospital mortality. Secondary outcomes were clinical improvement in relation to the WHO-CPS scale and need for intensive care unit admission. Clinical improvement was defined as at least 2 points reduction in the WHO-CPS score. Patients with less than 2 points reduction or without changes in their scores were identified as showing no improvement. Worsening clinical status was defined as elevation of the WHO-CPS score. Patients lost from follow-up were not included in the clinical outcome analyses. Outcomes were assessed on day 28. Subgrouping was performed according to age groups, where elderly patients were defined as patients at least or older than 65 years of age. Continuous variables were expressed as median ± interquartile range (IQR), with minimum–maximum ranges. Normality was tested by the Shapiro–Wilk test. Categorical variables were expressed as absolute numbers (*n*) with relative percentages (%). Statistical comparisons were done with Mann–Whitney *U*-test or Fisher’s exact test, depending on variable type. For all statistical tests, a 2-tailed *p*-value of < 0.05 determined statistical significance. To assess clinical improvement as a time-dependent outcome during the follow-up period, Kaplan–Meier probability curves of the subgroups were and statistically compared by the log rank test. Data collection was done with Microsoft Office Excel 2016; tests were calculated using IBM SPSS Statistics 23. For reporting, we adhere to the *Strengthening the Reporting of Observational Studies in Epidemiology* (STROBE) Statement.

## Results

From 3598 patients screened, 180 (5.0%) received CCP therapy during the study period and were included in the final cohort. Baseline demographic and clinical characteristics are detailed in Table 1. Four patients of the non-elderly group lost from follow-up. In the cohort, the median age was 63 ± 25 (18–96) years, with a male predominance (66.1%). The elderly group consisted of 83 patients, the non-elderly group of 97 patients. Main comorbidities were essential hypertension (51.1%), active haematological malignancy (43.3%) and chronic systemic corticosteroid or immunosuppressive treatment (38.9%); 7 chronic comorbidities were prevalent among elderly patients in a statistically significant manner. Fifty patients (27.8%) had mild, 57 (31.7%) had moderate and 73 (40.6%) had severe COVID-19, and while moderate-to-severe forms were more frequently diagnosed among elderly patients (25.8% and 39.2% vs. 38.6% and 42.2%, *p* = 0.04), the presence of pulmonary infiltrates consistent with COVID-19 on chest X-ray or CT was balanced between subgroups (84.5% vs. 78.3%, *p* = 0.28). The median to CCP from admission was 2 ± 4 (0–44) days, while the median CCP units administered was 2 ± 2 (1–8). Among other therapies against COVID-19 applied, only the use of remdesivir differed between subgroups (95.9% vs. 77.1%, *p* < 0.01).

**Table 1:** Baseline clinical characteristics of adult COVID-19 in-patients receiving COVID-19 convalescent plasma therapy, grouped by age (elderly subgroup defined as older than 65 years of age at study baseline)

Parameter	Total (*n* = 180)	Non-elderly (*n* = 97)	Elderly (*n* = 83)	*p* value
Age (years, median ± IQR, min–max)	63 ± 25 (16–96)	51 ± 15 (18–64)	77 ± 12 (65–96)	** < 0.01**
Male gender (*n*, %)	119 (66.1)	72 (74.2)	47 (56.6)	**0.01**
Comorbidities (*n*, %):				
- Essential hypertension	92 (51.1)	33 (34.0)	59 (71.1)	** < 0.01**
- Chronic heart disease	37 (20.6)	9 (9.3)	28 (33.7)	** < 0.01**
- Chronic vascular disease (excluding chronic neurovascular disease)	45 (25.0)	8 (8.3)	37 (44.6)	** < 0.01**
- Chronic neurovascular disease	30 (16.7)	2 (2.1)	28 (33.7)	** < 0.01**
- Chronic pulmonary disease	17 (9.4)	5 (5.3)	12 (14.5)	** < 0.01**
- Chronic liver disease	9 (5.0)	6 (6.2)	3 (3.6)	0.43
- Chronic kidney disease	35 (19.4)	9 (9.3)	26 (31.3)	** < 0.01**
- Diabetes mellitus	41 (22.8)	10 (10.3)	31 (37.6)	** < 0.01**
- Active oncological malignancy	5 (2.8)	1 (1.0)	4 (4.8)	0.12
- Active haematological malignancy	78 (43.3)	48 (49.5)	30 (36.1)	0.07
- Chronic systemic corticosteroid or immunosuppressive treatment	70 (38.9)	42 (43.3)	28 (33.7)	0.19
- Systemic autoimmune disease	9 (5.0)	6 (6.2)	3 (3.6)	0.43
- Pregnancy	2 (1.1)	2 (2.1)	0 (0)	n.a
Number of comorbidities per patient (median ± IQR, min–max)	2 ± 3 (0–7)	2 ± 2 (0–7)	4 ± 3 (0–7)	** < 0.01**
COVID-19 severity at baseline (*n*, %):				
- Mild disease (WHO-CPS 0–4)	50 (27.8)	34 (35.1)	16 (19.3)	**0.04**
- Moderate disease (WHO-CPS 5)	57 (31.7)	25 (25.8)	32 (38.6)	
- Severe disease (WHO-CPS 6)	73 (40.6)	38 (39.2)	35 (42.2)	
Presence of pulmonary infiltrates consistent with COVID-19 on chest X-ray or CT at baseline (*n*, %)	147 (81.7)	82 (84.5)	65 (78.3)	0.28
COVID-19 vaccination status at baseline (*n*, %):				
- Unvaccinated	128 (71.1)	70 (72.2)	58 (69.9)	0.42
- Incomplete primary series	4 (2.2)	3 (3.1)	1 (1.2)	
- Primary series only	36 (20)	20 (20.6)	16 (19.3)	
- Booster vaccinated	12 (6.7)	4 (4.1)	8 (9.6)	
Antiviral therapies for COVID-19 during hospitalisation (*n*, %):				
- Hydroxychloroquine	0 (0)	0 (0)	0 (0)	n.a
- Lopinavir/ritonavir	0 (0)	0 (0)	0 (0)	n.a
- Favipiravir	34 (18.9)	19 (19.6)	15 (18.1)	0.89
- Remdesivir	157 (87.2)	93 (95.9)	64 (77.1)	** < 0.01**
Immunomodulatory therapies for COVID-19 during hospitalisation (*n*, %):				
- Dexamethasone	159 (88.3)	88 (90.7)	71 (85.5)	0.28
- Tocilizumab	8 (4.4)	7 (7.2)	1 (1.2)	0.05
- Baricitinib	52 (28.9)	33 (34.0)	19 (22.9)	0.10
- Ruxolitinib	4 (2.2)	2 (2.1)	2 (2.4)	0.87
- Intravenous immunoglobulin	21 (11.7)	15 (15.5)	6 (7.2)	0.08
Monoclonal antibody therapies for COVID-19 during hospitalisation (*n*, %):				
- Bamlanivimab	3 (1.7)	1 (1.0)	2 (2.4)	0.47
- Casirivimab/imdevimab	6 (3.3)	4 (4.1)	2 (2.4)	0.52
Experimental therapies for COVID-19 during hospitalisation* (*n*, %):				
- SARS-CoV-2-specific T cell therapy	2 (1.1)	2 (2.1)	0 (0)	n.a
Time to CCP from symptom onset (days, median ± IQR, min–max)	8 ± 8 (0–58)	9 ± 9 (0–58)	7 ± 7 (0–36)	0.27

^*^In the context of an ongoing clinical trial

*CCP*, COVID-19 convalescent plasma; *ICU*, intensive care unit; *IQR*, interquartile range; *LOS*, length of hospital stay; *n.a.*, not applicable; *WHO-CPS*, World Health Organisation Clinical Progression Scale

**Fig. 1:**
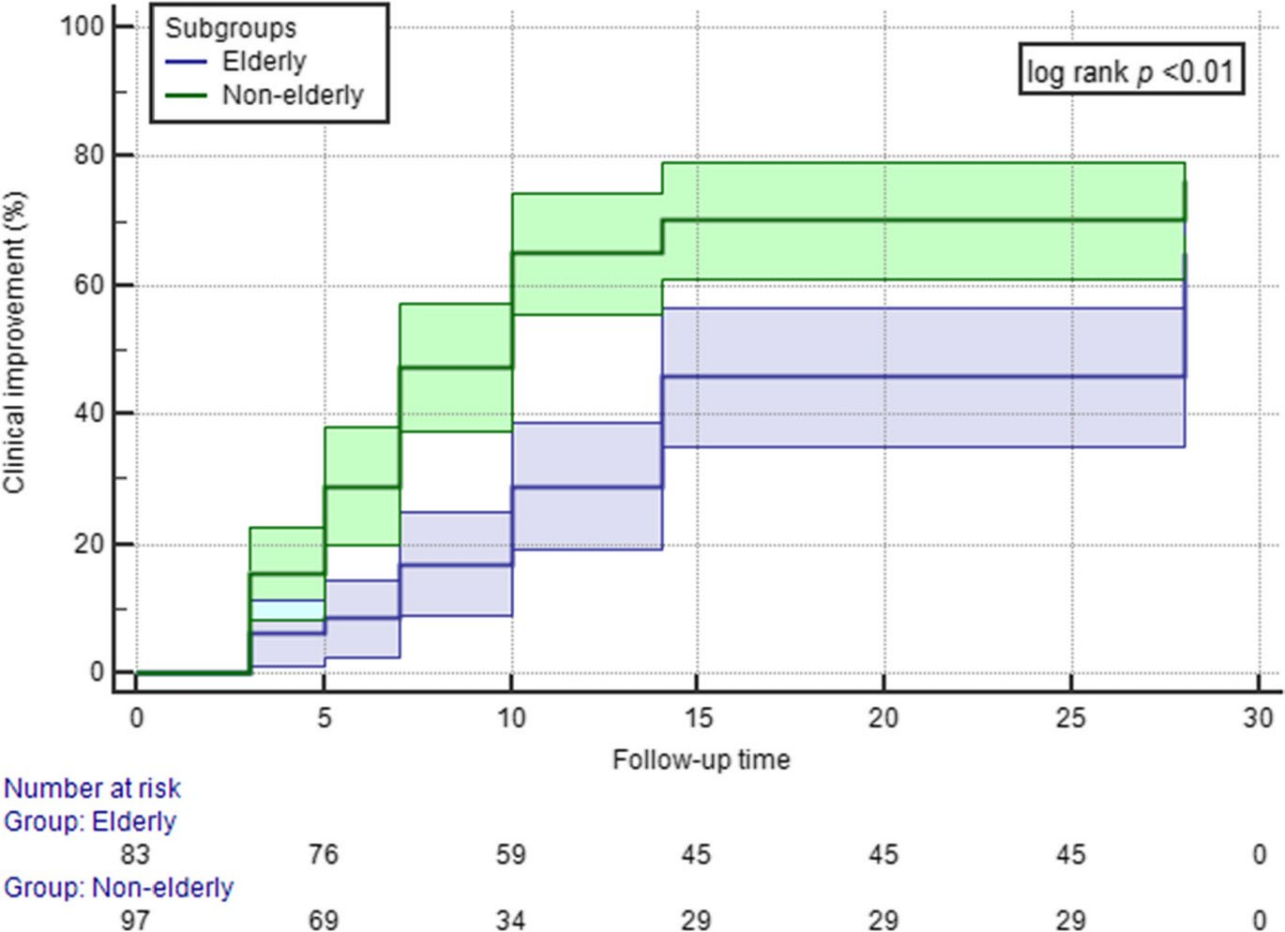
Kaplan–Meier curves for clinical improvement probability of adult COVID-19 in-patients receiving COVID-19 convalescent plasma therapy cumulated for 28 days, grouped by age (elderly subgroup defined as older than 65 years of age at study baseline)

Clinical outcomes are shown in Table 2. In-hospital all-cause mortality at 28 days was 11.4%, with a statistically significant difference between subgroups (3.2% vs. 20.5%, *p* < 0.01). Fifteen (8.5%) patients had to be transferred to the intensive care unit at 28 days, but rates were similar between elderly and non-elderly. Although the length of hospital stay was significantly longer among elderly patients, the length of ICU stay was similar between subgroups (14 ± 27 days vs. 5 ± 16 days, *p* = 0.1). In total, one hundred twenty-eight (72.7%) patients showed clinical improvement by day 28, with more favourable outcomes among non-elderly subgroup (79.6%, 16.1% and 4.3% vs. 65.1%, 13.3% and 21.7%, *p* < 0.01). In Fig. 1, probability distributions for clinical improvement in the non-elderly and elderly subgroups showed a statistical significant difference at 28 days (log rank *p* < 0.01).

**Table 2:** Clinical outcomes of adult COVID-19 in-patients receiving COVID-19 convalescent plasma therapy, grouped by age (elderly subgroup defined as older than 65 years of age at study baseline)

Outcome*	Total (*n* = 180)	Non-elderly (*n* = 97)	Elderly (*n* = 83)	*p* value
All-cause in-hospital mortality (*n*, %)	20 (11.4)	3 (3.2)	17 (20.5)	** < 0.01**
Need for ICU admission (*n*, %)	15 (8.5)	7 (7.5)	8 (9.6)	0.55
Clinical improvement according to WHO-CPS (*n*, %): - Improvement - No improvement - Worsening or death	128 (72.7) 26 (14.8) 22 (12.5)	74 (79.6) 15 (16.1) 4 (4.3)	54 (65.1) 11 (13.3) 18 (21.7)	** < 0.01**
Length of hospital stay (days, median ± IQR, min–max)	15 ± 14 (3**–**110)	14 ± 11 (3–110)	17 ± 16 (3–68)	**0.03**
Length of ICU stay (days, median ± IQR, min–max)	9 ± 22 (3**–**81)	14 ± 27 (7–81)	5 ± 16 (3–36)	0.1

^*^Patients lost to follow-up were not included in the final analysis

*ICU*, intensive care unit; *IQR*, interquartile range; *WHO-CPS*, World Health Organisation Clinical Progression Scale

## Discussion

### Main findings and limitations

Our main findings do not differ in merit from the data published in the literature so far. Our primary endpoint, in-hospital all-cause 28-day mortality (11.4%), was found to be similar to the 3.9 to 23% 28-day mortality rates of the CCP arms of RCTs studying similar numbers of hospitalised, moderate-to-severely ill COVID-19 patients [14–21]. The need for ICU admission (8.5%) was similar in our cohort compared to the findings of the two RCTs detailing rates of ICU admission and studying similar amounts of severely ill patients (8.3–15%) [15, 21]. However, in our cohort, among elderly versus patients of ≤ 65 years, a trend towards statistically similar rates of ICU admittance and lengths of ICU stay was noted. In this view, perhaps a residual benefit might be apprehended by delaying or possibly preventing transmission to the ICU in some elderly patients, preferably if convalescent plasma therapy is initiated early during the disease course.

Furthermore, considering statistically significant baseline differences between subgroups, namely that elderly patients had more comorbidities, presented with more severe disease and received remdesivir therapy in a lower rate, clinical outcomes might rather be related to these risk factors of unfavourable disease course, than to the ineffectiveness of CCP therapy.

Our study has several limitations. Due to its non-randomised structure and small sample size, our results must be interpreted with caution, despite the parallel findings with RCTs. The significant baseline characteristic differences and rates of remdesivir usage probably determined the results of our subgroup analysis results as well. As a placebo-controlled subgroup was not defined, an estimation of absolute risk reduction was not feasible. Lastly, some residual bias might have influenced our study data. Further investigation and determination whether defined patient subgroups would benefit from CCP therapy would be necessary.

### Previous studies from the literature

Several promising case reports and observational studies have been published, appreciating CCP therapy throughout the world, mainly during the first waves of the pandemic. Nevertheless, later published randomised controlled trials (RCT) could not universally reproduce these encouraging findings. Until the drafting of the current manuscript, 31 RCTs were published in the literature investigating the clinical effectiveness of CCP therapy in non-critically ill COVID-19 patients. The details, primary endpoints and results of these RCTs are summarised in Table 3. Eighteen (58.1%) of these trials were open label, and 5 of them were performed in an outpatient setting. Almost half of the studies, 14 (45.2%) enrolled at least 200 patients. Twenty-six (83.9%) enrolled patients with severe disease; 8 enrolled (25.8%) critically ill COVID-19 patients among others. Twelve RCTs were terminated earlier than planned, mainly due to decrease in numbers of enrollable patients or detecting potent neutralising antibody titres of patients comparable to the CCP products.

**Table 3:** Summary of the randomised controlled trials investigating the clinical effectiveness of CCP therapy in non-critically ill COVID-19 patients published, as of 15 October 2022

First author, journal and year of publication	Area	Trial design	Patient no. (CCP vs. SOC/placebo)	Age	Study setting	COVID-19 WHO severity	Transfusion timing from symptom onset (days)	Units and CCP titre transfused	Concomitant medications	Primary endpoints	Primary endpoint results
Li et al. JAMA, 2020 [23]	Wuhan, China	OL MC	23 vs. 22	70 ± 16^a^	Hospitalised	Severe, critical	30 ± 19^a^	NA units high titre	LPV/r, OST, ARB, RBV, CS, hIVIG, INF	Time to clinical improvement at 28 days	No significant difference, signal of possible clinical benefit for CCP among patients with severe COVID-19^1^
Agarwal et al. BMJ, 2020 (PLACID trial) [14]	India	OL MC	235 vs. 229	52 ± 19^a^	Hospitalised	Moderate, severe	8 ± 5^a^	2 × 1 units variable titre	HCQ, REM, LPV/r, OST, CS, TOZ, hIVIG	Progression to severe disease or death at 28 days	No significant difference
Simonovich et al. N Eng J Med, 2020 (PlasmAr trial) [15]	Argentina	DB MC PC	228 vs. 105	62 ± 20^a^	Hospitalised	Severe	8 ± 5^a^	1 × 2 units high titre	CS, LPV/r, TOZ, IVM, HCQ	Clinical status at 30 days	No significant difference
Libster et al. N Eng J Med, 2021 [25]	Argentina	DB PC	80 vs. 80	77.2 ± 8.6^b^	Outpatient hospitalised	Mild	< 3	1 × 1 unit high titre	-	Development of severe respiratory disease at 15 days	CCP significantly reduced progression risk to severe disease by 48%, CCP group showed longer time to development of severe disease^1^
Balcells et al. PLoS Med, 2021 [29]	Chile	OL SC	28 early vs. 30 deferred CCP	65.8 ± 65^b^	Hospitalised	Severe	6 ± 3^a^	2 × 1 units high titre	HCQ, CS, TOZ, LPV/r	Composite of hospitalisation > 14 days, IMV or in-hospital death	No significant difference
Pouladzadeh et al. Intern Emerg Med, 2021 [27]	Iran	SB SC	30 vs. 30	53.5 ± 10.3^b^ (CCP arm)	Hospitalised	Severe	< 7	1–2 × 1 unit variable titre	CHQ, LPV/r	Improvement of cytokine storm levels	Mean levels of lymphocytes and IL-10 significantly increased, levels of IL-6, TNF-α, and IFN-γ decreased in the CCP group
AlQahtani et al. Sci Rep, 2021 [30]	Bahrain	OL MC	20 vs. 20	52.6 ± 14.9^b^ (CCP arm)	Hospitalised	Severe	NA	2 × 1 units variable titre	HCQ, LPV/r, RBV, IFN, TOZ, CS	Requirement of NIV or IMV, duration of ventilation	No significant difference
RECOVERY Coll. Group JAMA, 2021 [28]	UK	OL MC	5795 vs. 5763	63.5 ± 14.7^b^	Hospitalised	Moderate, severe, critical	9 ± 6^a^	2 × 1 units high titre	LPV/r, CS, HCQ, REM, TOZ, SAR	All-cause mortality at 28 days	No significant difference
Gharbharan et al. Nat Commun, 2021 (ConCOVID trial) [31]	Netherlands	OL MC	43 vs. 43	63 ± 18^a^	Hospitalised	Severe	10 ± 9^a^	1 × 1 unit high titre	HCQ, LPV/r, REM	All-cause mortality at 60 days	No significant difference^2^
O’Donnell et al. J Clin Invest, 2021 [24]	USA, Brazil	DB MC	150 vs. 73	61^a^	Hospitalised	Severe, critical	9^a^	1 × 1 unit high titre	HCQ, REM, CS	Clinical status at 28 days	No significant difference, trends toward improved clinical status among patients who received CCP ≤ 7 days of symptom onset, CCP with higher titres of neutralising antibody and concomitant CS
Bennett-Guerrero et al. Crit Care Med, 2021 [32]	USA	DB SC	59 vs. 15	67 ± 15.8^b^ (CCP arm)	Hospitalised	Severe, critical	9 ± 12^a^ (CCP arm)	1 × 2 units high titre	HCQ, CS, REM, TOZ, SAR	Ventilator-free days at 28 days	No significant difference^3^
Kirenga et al. BMJ Open Resp Res, 2021 [16]	Uganda	OL SC	69 vs. 67	50 ± 23.5^a^	Hospitalised	Mild, severe	7 ± 4^a^	1 × 1 unit variable titre	CS	Time to viral clearance	No significant difference
Korley et al. N Eng J Med, 2021 (C3PO trial) [33]	USA	SB MC PC	257 vs. 254	54 ± 21^a^	Outpatient	Mild	4 ± 3^a^	1 × 1 unit high titre	-	Disease progression at 15 days	No significant difference^4^
Bégin et al. Nat Med, 2021 (CONCOR-1 trial) [17]	Canada, USA, Brazil	OL MC	625 vs. 313	69 ± 21^a^	Hospitalised	Severe	8 ± 5^a^	2 × 1 units high titre	CS	Intubation or death at 30 days	No significant difference^4^
Avendano-Sola et al. J Clin Invest, 2021 (ConPlas-19 trial) [19]	Spain	OL MC	179 vs. 171	62 ± 22^a^	Hospitalised	Severe	6 ± 3^a^	1 × 1 unit variable titre	REM, TOZ, CS	Proportion of patients in WHO ordinal categories of 5–7 at 14 days	No significant difference
Körper et al. J Clin Invest, 2021 (CAPSID trial) [34]	Germany	OL MC	53 vs. 52	60 ± 13^a^	Hospitalised	Severe, critical	7 ± 7^a^ (CCP arm)	3 × 1 units variable titre	REM, TOZ, CS	Composite of survival and no IMV, ICU or tachypnea at 21 days	No significant difference
Menichetti et al. JAMA Netw Open, 2021 (TSUNAMI trail) [19]	Italy	OL MC	241 vs. 246	64 ± 20^a^	Hospitalised	Moderate, severe	7.7 ± 4^a^	1–3 units high titre	REM, CS	Composite of worsening respiratory failure or death at 30 days	No significant difference
Holm et al. BMC Res Notes, 2021 [35]	Sweden	OL MC	17 vs. 14	80 ± 26^a^ (CCP)	Hospitalised	Severe	7 ± 4^a^ (CCP, arm)	3 × 1 units variable titre	CS, REM	Days with oxygen support at 28 days	No significant difference^5^
Bar et al. J Clin Invest, 2021 (PennCCP2 trial) [22]	USA	OL MC	41 vs. 39	63 ± 22^a^	Hospitalised	Severe	6 ± 5^a^	1 × 2 units variable titre	HCQ, CS, REM	Comparison of clinical severity scores	Significant difference favouring CCP
Baldeón et al. Transfus Med, 2022 [20]	Ecuador	DB MC PC	63 vs. 95	56.3 ± 12.7^b^ (CCP)	Hospitalised	Severe	10.6 ± 4.9^b^ (CCP arm)	1 × 1 unit variable titre	CS	Survival rate at 28 days	No significant difference^2^
Ray et al. Nat Commun, 2022 [36]	India	OL SC	40 vs. 40	NA	Hospitalised	Severe	4.2 ± 2.2^b^ (CCP arm)	2 × 1 units variable titre	HCQ, CS, REM, IVM	All-cause mortality at 30 days, identification of immunological correlates of response	No significant difference
De Santis et al. Emerg Infect Dis, 2022 [37]	Brazil	OL MC	36 vs. 71	60 ± 56^a^	Hospitalised	Severe, critical	8 ± 3^a^	3 × 1 units high titre	N/A	death rate at 30 and 60 days	no significant difference^1^
Ortigoza et al. JAMA Intern Med, 2022 (CONTAIN COVID-19 trial) [38]	USA	DB MC PC	468 vs. 465	63 ± 21^a^	Hospitalised	Severe	7 ± 5^a^	1 × 1 unit variable titre	HCQ, REM, CS	Clinical status at 14 days	No significant difference^1^
Alemany et al. Lancet Respir Med, 2022 (CONV-ERT trial) [39]	Spain	DB MC PC	188 vs. 188	56 ± 10^a^	Outpatient	Mild, moderate	4.4 ± 1.4^b^	1 × 1 unit high titre	N/A	Incidence of hospitalisation at 28 days, change in viral load in nasal swabs at 7 days	No significant difference
Devos et al. Eur Respir J, 2022 (DAWn-plasma trial) [21]	Belgium	OL MC	326 vs. 163	62 ± 14^b^	Hospitalised	Severe	6 ± 6^a^	2 × 2 units high titre	CHQ, HCQ, REM, TOZ, LPV/r, CS	Proportion of patients alive without IMV at 15 days	No significant difference
Sekine et al. Eur Resp J, 2022 (PLACOVID trial) [40]	Brazil	OL SC	80 vs. 80	60.5 ± 20^a^	Hospitalised	Severe, critical	10 ± 4^a^	2 × 1 units variable titre	CS	Proportion of patients with clinical improvement at 28 days	No significant difference
van den Berg et al Sci Rep, 2022 (PROTECT-Patient trial) [41]	South Africa	DB MC PC	52 vs. 51	56 ± 17^a^	Hospitalised	Severe	9 ± 5^a^	1 × 1 unit variable titre	CS	Successful treatment at 28 days	No significant difference^5^
Bajpai et al. BMJ Open, 2022 [42]	India	OL MC	200 vs. 200	55.5 ± 1.17^b^	Hospitalised	Severe, critical	NA	2 × 1 unit variable titre	REM, CS	Time to clinical improvement at 28 days	No significant difference
Sullivan et al. N Eng J Med, 2022 [26]	USA	DB MC	592 vs. 589	42 ± 22.5^a^	Outpatient	Mild	6 ± 3^a^	1 × 1 unit high titre	-	COVID-19-related hospitalisations at 28 days	CCP decreased the incidence of hospitalisation (RRR 54%)
Rojas et al. BMC Infect Dis, 2022 [43]	Colombia	SB MC	45 vs. 46	55.5 ± 24.8^a^ (CCP)	Hospitalised	Severe	10 ± 3^a^	2 × 1 unit variable titre	CS	Reduction of viral load, increase in titres of IgG and IgA	No significant difference, CCP was associated with an early transient increase in IgG levels at day 4
Gharbharan et al. Clin Microbiol Infect, 2022 (CoV-Eartly trial) [44]	Netherlands	DB MC PC	207 vs 209	med 60 ± 10^a^	Outpatient	Mild	med 5 ± 2^a^	1 × 1 unit variable titre	-	Clinical improvement at 28 days^6^	No significant difference

^a^Median ± interquartile range

^b^Mean ± standard deviation

^1^Early study termination due to decreasing number of enrolled patients

^2^Early study termination due to patients having potent neutralising antibody titres

^3^Early study termination due to emergency-use authorisation (EUA)

^4^Early study termination due to interim analysis indication

^5^Early study termination due to novel evidence published

^6^Recruitment discontinued due to elevating vaccination uptake rates

*ARB*, arbidol; *CCP*, COVID-19 convalescent plasma; *CHQ*, chloroquine; *COVID-19*, coronavirus disease 2019; *CS*, corticosteroid; *DB*, double blind; *HCQ*, hydroxychloroquine; *hIVIG*, human intravenous immunoglobulin; *ICU*, intensive care unit; *IFN-γ*, interferon gamma; *IgA*, immunoglobulin A; *IgG*, immunoglobulin G; *IL-10*, interleukin-10; *IL-6*, interleukin-6; *IMV*, invasive mechanical ventilation; *INF*, interferon; *IVM*, ivermectin; *LPV/r*, lopinavir/ritonavir; *MC*, multicentre; *NIV*, non-invasive ventilation; *OL*, open label; *OST*, oseltamivir; *PC*, placebo controlled; *RBV*, ribavirin; *REM*, remdesivir; *RRR*, relative risk reduction; *SAR*, sarilumab; *SB*, single blind; *SOC*, standard of care; *TNF-α*, tumour necrosis factor alpha; *TOZ*, tocilizumab; *WHO*, World Health Organisation

Twenty-three RCTs marked varieties of clinical status, progression or improvement as primary endpoint, but only one of them, carried out by Bar et al. in the USA, showed significant difference favouring CCP therapy among hospitalised patients [22]. One found signal of possible clinical benefit among patients with severe COVID-19 [23], and one other observed trends toward improvement in clinical status by day 28 among hospitalised patients receiving CCP within 7 days of symptom onset and with higher titres of neutralising antibodies and concomitant corticosteroid usage [24]. Among the RCTs evaluating CCP therapy with clinical primary endpoints in the outpatient setting, the one carried out by Libster et al., administering CCP less than 72 h after symptom onset in long-term care facilities in Argentina, found a statistically significant difference: CCP reduced the risk of progression to severe respiratory disease by 48%, and the CCP group showed longer time to the development to severe respiratory disease [25]. Sullivan et al. found that CCP decreased the incidence of hospitalisation when administered in a relatively younger outpatient population with mild disease in the USA; the relative risk reduction was 54% [26].

Nine RCTs included mortality in the primary endpoints, and none of them found significant difference in mortality rates among hospitalised COVID-19 patients. One study evaluated the cytokine storm indices after CCP therapy, with results that the mean levels of lymphocytes and IL-10 significantly increased, the levels of IL-6, TNF-α and IFN-γ decreased in the CCP group [27]. None of the RCTs enrolling more than 200 hospitalised patients showed significant differences in the primary endpoints. The largest RCT, as part of the RECOVERY trial, examined more than 11,000 patients with various disease severity, receiving CCP, but found no statistically significant difference between the CCP and usual care groups concerning mortality, length of hospital stay or progression to need for mechanical ventilation [28].

In summary, CCP therapy seems to have limited effect on clinical status and mortality based on the RCTs available in the literature. Favourable outcomes were seen mainly in outpatient settings, when CCP was applied very early in disease courses and in mild cases. Thus, RCTs published so far might have limitations as well. Many of them studied patients with heterogeneous disease severity, applied CCP units with various neutralisation antibody titres, determined different study endpoints and differed in the timing of CCP administration as well. Despite the heterogeneity, a meta-analysis of eligible RCTs found no correlation of CCP therapy with better clinical outcomes [45]. It could be hypothesised that CCP therapy might possess a clinically relevant effect in some COVID-19 patient subgroups. Elderly patients may benefit from CCP therapy, as they are expected to mount a slower antibody response due to immunosenescence. Also, patients with advanced B-cell defects or other immunocompromised conditions lacking efficient antibody producing ability may benefit from passive immunisation [46–50]. Furthermore, the Association for the Advancement of Blood and Biotherapies recently published a clinical practice guideline for the appropriate use of CCP that suggests CCP transfusion in addition to the usual standard of care for selected patient groups, based on low-to-moderate-certainty pieces of evidence [51].

## Conclusion

In our single-centre, open-label, prospective, observational study enrolling 180 patients, we found that among elderly patients hospitalised for non-critical COVID-19, the use of convalescent plasma therapy did not seem to possess an additional positive effect on most clinically relevant outcomes. Further investigation is needed to determine potential patient subgroups which might benefit from adequately timed CCP therapy.

## References

[1] Bartoletti M, Azap O, Barac A, Bussini L, Ergonul O, Krause R, Paño-Pardo JR, Power NR, Sibani M, Szabo BG, Tsiodras S, Verweij PE, Zollner-Schwetz I, Rodríguez-Baño J (2022). ESCMID COVID-19 living guidelines: drug treatment and clinical management. Clin Microbiol Infect.

[2] Zhou B, Zhong N, Guan Y (2007). Treatment with convalescent plasma for influenza A (H5N1) infection. N Engl J Med.

[3] Yu H, Gao Z, Feng Z, Shu Y, Xiang N, Zhou L, Huai Y, Feng L, Peng Z, Li Z, Xu C, Li J, Hu C, Li Q, Xu X, Liu X, Liu Z, Xu L, Chen Y, Luo H, Wei L, Zhang X, Xin J, Guo J, Wang Q, Yuan Z, Zhou L, Zhang K, Zhang W, Yang J, Zhong X, Xia S, Li L, Cheng J, Ma E, He P, Lee SS, Wang Y, Uyeki TM, Yang W (2008). Clinical characteristics of 26 human cases of highly pathogenic avian influenza A (H5N1) virus infection in China. PLoS ONE.

[4] Hung IFN, To KKW, Lee CK, Lee KL, Yan WW, Chan K, Chan WM, Ngai CW, Law KI, Chow FL, Liu R, Lai KY, Lau CCY, Liu SH, Chan KH, Lin CK, Yuen KY (2013). Hyperimmune IV immunoglobulin treatment: a multicenter double-blind randomized controlled trial for patients with severe 2009 influenza A(H1N1) infection. Chest.

[5] Cheng Y, Wong R, Soo YO, Wong WS, Lee CK, Ng MH, Chan P, Wong KC, Leung CB, Cheng G (2005). Use of convalescent plasma therapy in SARS patients in Hong Kong. Eur J Clin Microbiol Infect Dis.

[6] Soo YO, Cheng Y, Wong R, Hui DS, Lee CK, Tsang KK, Ng MH, Chan P, Cheng G, Sung JJ (2004). Retrospective comparison of convalescent plasma with continuing high-dose methylprednisolone treatment in SARS patients. Clin Microbiol Infect.

[7] Ko JH, Seok H, Cho SY, Ha YE, Baek JY, Kim SH, Kim YJ, Park JK, Chung CR, Kang ES, Cho D, Müller MA, Drosten C, Kang CI, Chung DR, Song JH, Peck KR (2018). Challenges of convalescent plasma infusion therapy in Middle East respiratory coronavirus infection: a single centre experience. Antivir Ther.

[8] van Griensven J, Edwards T, de Lamballerie X, Semple MG, Gallian P, Baize S, Horby PW, Raoul H, Magassouba N, Antierens A, Lomas C, Faye O, Sall AA, Fransen K, Buyze J, Ravinetto R, Tiberghien P, Claeys Y, De Crop M, Lynen L, Bah EI, Smith PG, Delamou A, De Weggheleire A, Haba N (2016). Evaluation of convalescent plasma for Ebola virus disease in Guinea. N Engl J Med.

[9] Sahr F, Ansumana R, Massaquoi TA, Idriss BR, Sesay FR, Lamin JM, Baker S, Nicol S, Conton B, Johnson W, Abiri OT, Kargbo O, Kamara P, Goba A, Russell JB, Gevao SM (2017). Evaluation of convalescent whole blood for treating Ebola virus disease in Freetown, Sierra Leone. J Infect.

[10] Garraud O, Heshmati F, Pozzetto B, Lefrere F, Girot R, Saillol A, Laperche S (2016). Plasma therapy against infectious pathogens, as of yesterday, today and tomorrow. Transfus Clin Biol.

[11] Mair-Jenkins J, Saavedra-Campos M, Baillie JK, Cleary P, Khaw FM, Lim WS, Makki S, Rooney KD, Nguyen-Van-Tam JS, Beck CR (2015). The effectiveness of convalescent plasma and hyperimmune immunoglobulin for the treatment of severe acute respiratory infections of viral etiology: a systematic review and exploratory meta-analysis. J Infect Dis.

[12] Rojas M, Rodríguez Y, Monsalve DM, Acosta-Ampudia Y, Camacho B, Gallo JE, Rojas-Villarraga A, Ramírez-Santana C, Díaz-Coronado JC, Manrique R, Mantilla RD, Shoenfeld Y, Anaya JM (2020). Convalescent plasma in Covid-19: possible mechanisms of action. Autoimmun Rev.

[13] Ramaswamy P, Gong JJ, Saleh SN, McDonald SA, Blumberg S, Medford RJ, Liu X. Developing a COVID-19 WHO Clinical Progression Scale inpatient database from electronic health record data. J Am Med Inform Assoc. 2022;14 29(7):1279–1285. 10.1093/jamia/ocac041 10.1093/jamia/ocac041 PMC9196693 35289912

[14] Agarwal A, Mukherjee A, Kumar G, Chatterjee P, Bhatnagar T, Malhotra P (2020). Convalescent plasma in the management of moderate COVID-19 in adults in India: open label phase II multicentre randomised controlled trial (PLACID Trial). BMJ.

[15] Simonovich VA, Burgos Pratx LD, Scibona P, Beruto MV, Vallone MG, Vázquez C, Savoy N, Giunta DH, Pérez LG, Sánchez MDL, Gamarnik AV, Ojeda DS, Santoro DM, Camino PJ, Antelo S, Rainero K, Vidiella GP, Miyazaki EA, Cornistein W, Trabadelo OA, Ross FM, Spotti M, Funtowicz G, Scordo WE, Losso MH, Ferniot I, Pardo PE, Rodriguez E, Rucci P, Pasquali J, Fuentes NA, Esperatti M, Speroni GA, Nannini EC, Matteaccio A, Michelangelo HG, Follmann D, Lane HC, Belloso WH (2021). A randomized trial of convalescent plasma in COVID-19 severe pneumonia. N Engl J Med.

[16] Kirenga B, Byakika-Kibwika P, Muttamba W, Kayongo A, Loryndah NO, Mugenyi L, Kiwanuka N, Lusiba J, Atukunda A, Mugume R, Ssali F, Ddungu H, Katagira W, Sekibira R, Kityo C, Kyeyune D, Acana S, Aanyu-Tukamuhebwa H, Kabweru W, Nakwagala F, Bagaya BS, Kimuli I, Nantanda R, Buregyeya E, Byarugaba B, Olaro C, Mwebesa H, Joloba ML, Siddharthan T, Bazeyo W. Efficacy of convalescent plasma for treatment of COVID-19 in Uganda. BMJ Open Respir Res. 2021;8(1):e001017. 10.1136/bmjresp-2021-001017 10.1136/bmjresp-2021-001017 PMC8354811 34376401

[17] Bégin P, Callum J, Jamula E, Cook R, Heddle NM, Tinmouth A, Zeller MP, Beaudoin-Bussières G, Amorim L, Bazin R, Loftsgard KC, Carl R, Chassé M, Cushing MM, Daneman N, Devine DV, Dumaresq J, Fergusson DA, Gabe C, Glesby MJ, Li N, Liu Y, McGeer A, Robitaille N, Sachais BS, Scales DC, Schwartz L, Shehata N, Turgeon AF, Wood H, Zarychanski R, Finzi A, Arnold DM (2021). Convalescent plasma for hospitalized patients with COVID-19: an open-label, randomized controlled trial. Nat Med.

[18] Avendaño-Solá C, Ramos-Martínez A, Muñez-Rubio E, Ruiz-Antorán B, Malo de Molina R, Torres F, Fernández-Cruz A, Calderón-Parra J, Payares-Herrera C, Díaz de Santiago A, Romera-Martínez I, Pintos I, Lora-Tamayo J, Mancheño-Losa M, Paciello ML, Martínez-González AL, Vidán-Estévez J, Nuñez-Orantos MJ, Saez-Serrano MI, Porras-Leal ML, Jarilla-Fernández MC, Villares P, de Oteyza JP, Ramos-Garrido A, Blanco L, Madrigal-Sánchez ME, Rubio-Batllés M, Velasco-Iglesias A, Paño-Pardo JR, Moreno-Chulilla JA, Muñiz-Díaz E, Casas-Flecha I, Pérez-Olmeda M, García-Pérez J, Alcamí J, Bueno JL, Duarte RF, ConPlas-19 Study Group. A multicenter randomized open-label clinical trial for convalescent plasma in patients hospitalized with COVID-19 pneumonia. J Clin Invest. 2021;131(20):e152740. 10.1172/jci152740 10.1172/JCI152740 PMC8516461 34473652

[19] Menichetti F, Popoli P, Puopolo M, Spila Alegiani S, Tiseo G, Bartoloni A, De Socio GV, Luchi S, Blanc P, Puoti M, Toschi E, Massari M, Palmisano L, Marano G, Chiamenti M, Martinelli L, Franchi S, Pallotto C, Suardi LR, Luciani Pasqua B, Merli M, Fabiani P, Bertolucci L, Borchi B, Modica S, Moneta S, Marchetti G, d’Arminio Monforte A, Stoppini L, Ferracchiato N, Piconi S, Fabbri C, Beccastrini E, Saccardi R, Giacometti A, Esperti S, Pierotti P, Bernini L, Bianco C, Benedetti S, Lanzi A, Bonfanti P, Massari M, Sani S, Saracino A, Castagna A, Trabace L, Lanza M, Focosi D, Mazzoni A, Pistello M, Falcone M, TSUNAMI Study group. Effect of high-titer convalescent plasma on progression to severe respiratory failure or death in hospitalized patients with COVID-19 pneumonia: a randomized clinical trial. JAMA Netw Open. 2021;4(11):e2136246. 10.1001/jamanetworkopen.2021.36246 10.1001/jamanetworkopen.2021.36246 PMC8630572 34842924

[20] Baldeón ME, Maldonado A, Ochoa-Andrade M, Largo C, Pesantez M, Herdoiza M, Granja G, Bonifaz M, Espejo H, Mora F, Abril-López P, Armijo LKR, Pacheco V, Salazar R, Reinthaller S, Zertuche F, Fornasini M (2022). Effect of convalescent plasma as complementary treatment in patients with moderate COVID-19 infection. Transfus Med.

[21] Devos T, Van Thillo Q, Compernolle V, Najdovski T, Romano M, Dauby N, Jadot L, Leys M, Maillart E, Loof S, Seyler L, Moonen M, Moutschen M, Van Regenmortel N, Ariën KK, Barbezange C, Betrains A, Garigliany M, Engelen MM, Gyselinck I, Maes P, Schauwvlieghe A, Liesenborghs L, Belmans A, Verhamme P, Meyfroidt G; DAWn-plasma investigators. Early high antibody titre convalescent plasma for hospitalised COVID-19 patients: DAWn-plasma. Eur Respir J. 2022;10 59(2):2101724. 10.1183/13993003.01724-2021 10.1183/13993003.01724-2021 PMC8576805 34446469

[22] Bar KJ, Shaw PA, Choi GH, Aqui N, Fesnak A, Yang JB, Soto-Calderon H, Grajales L, Starr J, Andronov M, Mastellone M, Amonu C, Feret G, DeMarshall M, Buchanan M, Caturla M, Gordon J, Wanicur A, Monroy MA, Mampe F, Lindemuth E, Gouma S, Mullin AM, Barilla H, Pronina A, Irwin L, Thomas R, Eichinger RA, Demuth F, Luning Prak ET, Pascual JL, Short WR, Elovitz MA, Baron J, Meyer NJ, Degnan KO, Frank I, Hensley SE, Siegel DL, Tebas P (2021). A randomized controlled study of convalescent plasma for individuals hospitalized with COVID-19 pneumonia. J Clin Invest.

[23] Li L, Zhang W, Hu Y, Tong X, Zheng S, Yang J, Kong Y, Ren L, Wei Q, Mei H, Hu C, Tao C, Yang R, Wang J, Yu Y, Guo Y, Wu X, Xu Z, Zeng L, Xiong N, Chen L, Wang J, Man N, Liu Y, Xu H, Deng E, Zhang X, Li C, Wang C, Su S, Zhang L, Wang J, Wu Y, Liu Z (2020). Effect of convalescent plasma therapy on time to clinical improvement in patients with severe and life-threatening COVID-19: a randomized clinical trial. JAMA.

[24] O'Donnell MR, Grinsztejn B, Cummings MJ, Justman JE, Lamb MR, Eckhardt CM, Philip NM, Cheung YK, Gupta V, João E, Pilotto JH, Diniz MP, Cardoso SW, Abrams D, Rajagopalan KN, Borden SE, Wolf A, Sidi LC, Vizzoni A, Veloso VG, Bitan ZC, Scotto DE, Meyer BJ, Jacobson SD, Kantor A, Mishra N, Chauhan LV, Stone EF, Dei Zotti F, La Carpia F, Hudson KE, Ferrara SA, Schwartz J, Stotler BA, Lin WW, Wontakal SN, Shaz B, Briese T, Hod EA, Spitalnik SL, Eisenberger A, Lipkin WI. A randomized double-blind controlled trial of convalescent plasma in adults with severe COVID-19. J Clin Invest. 2021;131(13):e150646. 10.1172/jci150646 10.1172/JCI150646 PMC8245169 33974559

[25] Libster R, Pérez Marc G, Wappner D, Coviello S, Bianchi A, Braem V, Esteban I, Caballero MT, Wood C, Berrueta M, Rondan A, Lescano G, Cruz P, Ritou Y, Fernández Viña V, Álvarez Paggi D, Esperante S, Ferreti A, Ofman G, Ciganda Á, Rodriguez R, Lantos J, Valentini R, Itcovici N, Hintze A, Oyarvide ML, Etchegaray C, Neira A, Name I, Alfonso J, López Castelo R, Caruso G, Rapelius S, Alvez F, Etchenique F, Dimase F, Alvarez D, Aranda SS, Sánchez Yanotti C, De Luca J, Jares Baglivo S, Laudanno S, Nowogrodzki F, Larrea R, Silveyra M, Leberzstein G, Debonis A, Molinos J, González M, Perez E, Kreplak N, Pastor Argüello S, Gibbons L, Althabe F, Bergel E, Polack FP (2021). Early high-titer plasma therapy to prevent severe COVID-19 in older adults. N Engl J Med.

[26] Sullivan DJ, Gebo KA, Shoham S, Bloch EM, Lau B, Shenoy AG, Mosnaim GS, Gniadek TJ, Fukuta Y, Patel B, Heath SL, Levine AC, Meisenberg BR, Spivak ES, Anjan S, Huaman MA, Blair JE, Currier JS, Paxton JH, Gerber JM, Petrini JR, Broderick PB, Rausch W, Cordisco ME, Hammel J, Greenblatt B, Cluzet VC, Cruser D, Oei K, Abinante M, Hammitt LL, Sutcliffe CG, Forthal DN, Zand MS, Cachay ER, Raval JS, Kassaye SG, Foster EC, Roth M, Marshall CE, Yarava A, Lane K, McBee NA, Gawad AL, Karlen N, Singh A, Ford DE, Jabs DA, Appel LJ, Shade DM, Ehrhardt S, Baksh SN, Laeyendecker O, Pekosz A, Klein SL, Casadevall A, Tobian AAR, Hanley DF (2022). Early outpatient treatment for COVID-19 with convalescent plasma. N Engl J Med.

[27] Pouladzadeh M, Safdarian M, Eshghi P, Abolghasemi H, Bavani AG, Sheibani B, Moradi Choghakabodi P, Feghhi A, Ghafourian Boroujerdnia M, Forouzan A, Jalali Far MA, Kaydani GA, Rajaei E, Amin M, Torabizadeh M, Yousefi F, Hadaddezfuli R (2021). A randomized clinical trial evaluating the immunomodulatory effect of convalescent plasma on COVID-19-related cytokine storm. Intern Emerg Med.

[28] RECOVERY Collaborative Group (2021). Convalescent plasma in patients admitted to hospital with COVID-19 (RECOVERY): a randomised controlled, open-label, platform trial. Lancet.

[29] Balcells ME, Rojas L, Le Corre N, Martínez-Valdebenito C, Ceballos ME, Ferrés M, Chang M, Vizcaya C, Mondaca S, Huete Á, Castro R, Sarmiento M, Villarroel L, Pizarro A, Ross P, Santander J, Lara B, Ferrada M, Vargas-Salas S, Beltrán-Pavez C, Soto-Rifo R, Valiente-Echeverría F, Caglevic C, Mahave M, Selman C, Gazitúa R, Briones JL, Villarroel-Espindola F, Balmaceda C, Espinoza MA, Pereira J, Nervi B (2021). Early versus deferred anti-SARS-CoV-2 convalescent plasma in patients admitted for COVID-19: a randomized phase II clinical trial. PLoS Med.

[30] AlQahtani M, Abdulrahman A, Almadani A, Alali SY, Al Zamrooni AM, Hejab AH, Conroy RM, Wasif P, Otoom S, Atkin SL, Abduljalil M (2021). Randomized controlled trial of convalescent plasma therapy against standard therapy in patients with severe COVID-19 disease. Sci Rep.

[31] Gharbharan A, Jordans CCE, GeurtsvanKessel C, den Hollander JG, Karim F, Mollema FPN, Stalenhoef-Schukken JE, Dofferhoff A, Ludwig I, Koster A, Hassing RJ, Bos JC, van Pottelberge GR, Vlasveld IN, Ammerlaan HSM, van Leeuwen-Segarceanu EM, Miedema J, van der Eerden M, Schrama TJ, Papageorgiou G, Te Boekhorst P, Swaneveld FH, Mueller YM, Schreurs MWJ, van Kampen JJA, Rockx B, Okba NMA, Katsikis PD, Koopmans MPG, Haagmans BL, Rokx C, Rijnders BJA (2021). Effects of potent neutralizing antibodies from convalescent plasma in patients hospitalized for severe SARS-CoV-2 infection. Nat Commun.

[32] Bennett-Guerrero E, Romeiser JL, Talbot LR, Ahmed T, Mamone LJ, Singh SM, Hearing JC, Salman H, Holiprosad DD, Freedenberg AT, Carter JA, Browne NJ, Cosgrove ME, Shevik ME, Generale LM, Andrew MA, Nachman S, Fries BC (2021). Severe acute respiratory syndrome coronavirus 2 convalescent plasma versus standard plasma in coronavirus disease 2019 infected hospitalized patients in New York: a double-blind randomized trial. Crit Care Med.

[33] Korley FK, Durkalski-Mauldin V, Yeatts SD, Schulman K, Davenport RD, Dumont LJ, El Kassar N, Foster LD, Hah JM, Jaiswal S, Kaplan A, Lowell E, McDyer JF, Quinn J, Triulzi DJ, Van Huysen C, Stevenson VLW, Yadav K, Jones CW, Kea B, Burnett A, Reynolds JC, Greineder CF, Haas NL, Beiser DG, Silbergleit R, Barsan W, Callaway CW (2021). Early convalescent plasma for high-risk outpatients with COVID-19. N Engl J Med.

[34] Körper S, Weiss M, Zickler D, Wiesmann T, Zacharowski K, Corman VM, Grüner B, Ernst L, Spieth P, Lepper PM, Bentz M, Zinn S, Paul G, Kalbhenn J, Dollinger MM, Rosenberger P, Kirschning T, Thiele T, Appl T, Mayer B, Schmidt M, Drosten C, Wulf H, Kruse JM, Jungwirth B, Seifried E, Schrezenmeier H, CAPSID Clinical Trial Group. Results of the CAPSID randomized trial for high-dose convalescent plasma in patients with severe COVID-19. J Clin Invest. 2021;131(20):e152264. 10.1172/JCI152264 10.1172/JCI152264 PMC8516466 34464358

[35] Holm K, Lundgren MN, Kjeldsen-Kragh J, Ljungquist O, Böttiger B, Wikén C, Öberg J, Fernström N, Rosendal E, Överby AK, Wigren Byström J, Forsell M, Landin-Olsson M, Rasmussen M (2021). Convalescence plasma treatment of COVID-19: results from a prematurely terminated randomized controlled open-label study in Southern Sweden. BMC Res Notes.

[36] Ray Y, Paul SR, Bandopadhyay P, D’Rozario R, Sarif J, Raychaudhuri D, Bhowmik D, Lahiri A, Vasudevan JS, Maurya R, Kanakan A, Sharma S, Kumar M, Singh P, Roy R, Chaudhury K, Maiti R, Bagchi S, Maiti A, Perwez MM, Mondal A, Tewari A, Mandal S, Roy A, Saha M, Biswas D, Maiti C, Bhaduri R, Chakraborty S, Sarkar BS, Haldar A, Saha B, Sengupta S, Pandey R, Chatterjee S, Bhattacharya P, Paul S, Ganguly D (2022). A phase 2 single center open label randomised control trial for convalescent plasma therapy in patients with severe COVID-19. Nat Commun.

[37] De Santis GC, Oliveira LC, Garibaldi PMM, Almado CEL, Croda J, Arcanjo GGA, Oliveira ÉAF, Tonacio AC, Langhi DM, Bordin JO, Gilio RN, Palma LC, Santos EV, Haddad SK, Prado BPA, Pontelli MC, Gomes R, Miranda CH, Auxiliadora Martins M, Covas DT, Arruda E, Fonseca BAL, Calado RT (2022). High-dose convalescent plasma for treatment of severe COVID-19. Emerg Infect Dis.

[38] Ortigoza MB, Yoon H, Goldfeld KS, Troxel AB, Daily JP, Wu Y, Li Y, Wu D, Cobb GF, Baptiste G, O’Keeffe M, Corpuz MO, Ostrosky-Zeichner L, Amin A, Zacharioudakis IM, Jayaweera DT, Wu Y, Philley JV, Devine MS, Desruisseaux MS, Santin AD, Anjan S, Mathew R, Patel B, Nigo M, Upadhyay R, Kupferman T, Dentino AN, Nanchal R, Merlo CA, Hager DN, Chandran K, Lai JR, Rivera J, Bikash CR, Lasso G, Hilbert TP, Paroder M, Asencio AA, Liu M, Petkova E, Bragat A, Shaker R, McPherson DD, Sacco RL, Keller MJ, Grudzen CR, Hochman JS, Pirofski LA, Parameswaran L, Corcoran AT, Rohatgi A, Wronska MW, Wu X, Srinivasan R, Deng FM, Filardo TD, Pendse J, Blaser SB, Whyte O, Gallagher JM, Thomas OE, Ramos D, Sturm-Reganato CL, Fong CC, Daus IM, Payoen AG, Chiofolo JT, Friedman MT, Wu DW, Jacobson JL, Schneider JG, Sarwar UN, Wang HE, Huebinger RM, Dronavalli G, Bai Y, Grimes CZ, Eldin KW, Umana VE, Martin JG, Heath TR, Bello FO, Ransford DL, Laurent-Rolle M, Shenoi SV, Akide-Ndunge OB, Thapa B, Peterson JL, Knauf K, Patel SU, Cheney LL, Tormey CA, Hendrickson JE (2022). Efficacy and safety of COVID-19 convalescent plasma in hospitalized patients: a randomized clinical trial. JAMA Intern Med.

[39] Alemany A, Millat-Martinez P, Corbacho-Monné M, Malchair P, Ouchi D, Ruiz-Comellas A, Ramírez-Morros A, Rodríguez Codina J, Amado Simon R, Videla S, Costes G, Capdevila-Jáuregui M, Torrano-Soler P, San José A, Bonet Papell G, Puig J, Otero A, Ruibal Suarez JC, Zarauza Pellejero A, Llopis Roca F, Rodriguez Cortez O, Garcia Garcia V, Vidal-Alaball J, Millan A, Contreras E, Grifols JR, Ancochea À, Galvan-Femenia I, Piccolo Ferreira F, Bonet M, Cantoni J, Prat N, Ara J, Forcada Arcarons A, Farré M, Pradenas E, Blanco J, Àngel Rodriguez-Arias M, Fernández Rivas G, Marks M, Bassat Q, Blanco I, Baro B, Clotet B, Mitjà O; CONV-ERT Group. High-titre methylene blue-treated convalescent plasma as an early treatment for outpatients with COVID-19: a randomised, placebo-controlled trial. Lancet Respir Med. 2022;10(3):278–288. 10.1016/S2213-2600(21)00545-2 10.1016/S2213-2600(21)00545-2 PMC8828369 35150610

[40] Sekine L, Arns B, Fabro BR, Cipolatt MM, Machado RRG, Durigon EL, Parolo E, Pellegrini JAS, Viana MV, Schwarz P, Lisboa TC, Dora JMS, Portich JP, Paz AA, Silla L, Balsan AM, Schirmer FD, Franz JPM, da-Silveira LM, Breunig RC, Petersen V, Sosnoski M, Mesquita NF, Volpato FCZ, Sganzerla D, Falavigna M, Rosa RG, Zavascki AP; PLACOVID Study Group. Convalescent plasma for COVID-19 in hospitalised patients: an open-label, randomised clinical trial. Eur Respir J. 2022;10 59(2):2101471. 10.1183/13993003.01471-2021 10.1183/13993003.01471-2021 PMC8287736 34244316

[41] van den Berg K, Glatt TN, Vermeulen M, Little F, Swanevelder R, Barrett C, Court R, Bremer M, Nyoni C, Swarts A, Mmenu C, Crede T, Kritzinger G, Naude J, Szymanski P, Cowley J, Moyo-Gwete T, Moore PL, Black J, Singh J, Bhiman JN, Baijnath P, Mody P, Malherbe J, Potgieter S, van Vuuren C, Maasdorp S, Wilkinson RJ, Louw VJ, Wasserman S (2022). Convalescent plasma in the treatment of moderate to severe COVID-19 pneumonia: a randomized controlled trial (PROTECT-Patient Trial). Sci Rep.

[42] Bajpai M, Maheshwari A, Dogra V, Kumar S, Gupta E, Kale P, Saluja V, Thomas SS, Trehanpati N, Bihari C, Agarwal R, Bharti P, Shankar P, Hussain J, Chhabra K, Gupta A, Narayanan A, Agarwal S, Jain S, Bhardwaj A, Kumar G, Yadav BK, Sarin SK (2022). Efficacy of convalescent plasma therapy in the patient with COVID-19: a randomised control trial (COPLA-II trial). BMJ Open.

[43] Rojas M, Rodríguez Y, Hernández JC, Díaz-Coronado JC, Vergara JAD, Vélez VP, Mancilla JP, Araujo I, Yepes JT, Ricaurte OB, Pardo-Oviedo JM, Monsalve DM, Acosta-Ampudia Y, Ramírez-Santana C, García PG, Landinez LA, Correales LD, Grass JS, Pérez CR, López GS, Mateus N, Mancera L, Devia RR, Orjuela JE, Parra-Moreno CR, Buitrago AA, Ordoñez IE, Osorio CF, Ballesteros N, Patiño LH, Castañeda S, Muñoz M, Ramírez JD, Bastard P, Gervais A, Bizien L, Casanova JL, Camacho B, Gallo JE, Gómez O, Rojas-Villarraga A, Pérez CE, Manrique R, Mantilla RD, Anaya JM. Safety and efficacy of convalescent plasma for severe COVID-19: a randomized, single blinded, parallel, controlled clinical study. BMC Infect Dis. 2022;27 22(1):575. 10.1186/s12879-022-07560-7 10.1186/s12879-022-07560-7 PMC9235185 35761219

[44] Gharbharan A, Jordans C, Zwaginga L, Papageorgiou G, van Geloven N, van Wijngaarden P, den Hollander J, Karim F, van Leeuwen-Segarceanu E, Soetekouw R, Lammers J, Postma D, Kampschreur L, Groeneveld G, Swaneveld F, van der Schoot CE, Götz H, Haagmans B, Koopmans M, Bogers S, Geurtsvankessel C, Zwaginga JJ, Rokx C, Rijnders B; CoV-Early study group. Outpatient convalescent plasma therapy for high-risk patients with early COVID-19: a randomized placebo-controlled trial. Clin Microbiol Infect. 2022;S1198–743X(22)00421–9. 10.1016/j.cmi.2022.08.005 10.1016/j.cmi.2022.08.005 PMC9395229 36007870

[45] Troxel AB, Petkova E, Goldfeld K, Liu M, Tarpey T, Wu Y, Wu D, Agarwal A, Avendaño-Solá C, Bainbridge E, Bar KJ, Devos T, Duarte RF, Gharbharan A, Hsue PY, Kumar G, Luetkemeyer AF, Meyfroidt G, Nicola AM, Mukherjee A, Ortigoza MB, Pirofski LA, Rijnders BJA, Rokx C, Sancho-Lopez A, Shaw P, Tebas P, Yoon HA, Grudzen C, Hochman J, Antman EM (2022). Association of convalescent plasma treatment with clinical status in patients hospitalized with COVID-19: a meta-analysis. JAMA Netw Open.

[46] Thompson MA, Henderson JP, Shah PK, Rubinstein SM, Joyner MJ, Choueiri TK, Flora DB, Griffiths EA, Gulati AP, Hwang C, Koshkin VS, Papadopoulos EB, Robilotti EV, Su CT, Wulff-Burchfield EM, Xie Z, Yu PP, Mishra S, Senefeld JW, Shah DP, Warner JL (2021). Association of convalescent plasma therapy with survival in patients with hematologic cancers and COVID-19. JAMA Oncol.

[47] Hueso T, Pouderoux C, Péré H, Beaumont AL, Raillon LA, Ader F, Chatenoud L, Eshagh D, Szwebel TA, Martinot M, Camou F, Crickx E, Michel M, Mahevas M, Boutboul D, Azoulay E, Joseph A, Hermine O, Rouzaud C, Faguer S, Petua P, Pommeret F, Clerc S, Planquette B, Merabet F, London J, Zeller V, Ghez D, Veyer D, Ouedrani A, Gallian P, Pacanowski J, Mékinian A, Garnier M, Pirenne F, Tiberghien P, Lacombe K (2020). Convalescent plasma therapy for B-cell-depleted patients with protracted COVID-19. Blood.

[48] Betrains A, Godinas L, Woei AJF, Rosseels W, Van Herck Y, Lorent N, Dierickx D, Compernolle V, Meyfroidt G, Vanderbeke L, Vergote V, Lagrou K, Verhamme P, Wauters J, Vermeersch P, Devos T, Maes P, Vanderschueren S (2021). Convalescent plasma treatment of persistent severe acute respiratory syndrome coronavirus-2 (SARS-CoV-2) infection in patients with lymphoma with impaired humoral immunity and lack of neutralising antibodies. Br J Haematol.

[49] Gupta A, Kute VB, Patel HV, Engineer DP, Banerjee S, Modi PR, Rizvi SJ, Mishra VV, Patel AH, Navadiya V (2021). Feasibility of convalescent plasma therapy in kidney transplant recipients with severe COVID-19: a single-center prospective cohort study. Exp Clin Transplant.

[50] Senefeld JW, Klassen SA, Ford SK, Senese KA, Wiggins CC, Bostrom BC, Thompson MA, Baker SE, Nicholson WT, Johnson PW, Carter RE, Henderson JP, Hartman WR, Pirofski LA, Wright RS, Fairweather L, Bruno KA, Paneth NS, Casadevall A, Joyner MJ (2021). Use of convalescent plasma in COVID-19 patients with immunosuppression. Transfusion.

[51] Estcourt LJ, Cohn CS, Pagano MB, Iannizzi C, Kreuzberger N, Skoetz N, Allen ES, Bloch EM, Beaudoin G, Casadevall A, Devine DV, Foroutan F, Gniadek TJ, Goel R, Gorlin J, Grossman BJ, Joyner MJ, Metcalf RA, Raval JS, Rice TW, Shaz BH, Vassallo RR, Winters JL, Tobian AAR (2022). Clinical practice guidelines from the association for the advancement of blood and biotherapies (AABB): COVID-19 convalescent plasma. Ann Intern Med.

